# Epigenetic regulation of *IFITM1* expression in lipopolysaccharide-stimulated human mesenchymal stromal cells

**DOI:** 10.1186/s13287-019-1531-3

**Published:** 2020-01-07

**Authors:** Sun Hwa Kim, Hae In Choi, Mi Ran Choi, Ga Yeong An, Bert Binas, Kyoung Hwa Jung, Young Gyu Chai

**Affiliations:** 1 Department of Molecular & Life Science, Hanyang University, Ansan, 15588 Republic of Korea; 2 Department of Bionanotechnology, Hanyang University, Seoul, 04673 Republic of Korea; 3 Department of Psychiatry, The Catholic University of Korea, Seoul, 06591 Republic of Korea; 4 Convergence Technology Campus of Korea Polytechnic II, Incheon, 21417 Republic of Korea

**Keywords:** Mesenchymal stromal cells, Interferon-induced transmembrane protein 1, Toll-like receptor 4, Lipopolysaccharide, Enhancer, NF-κB

## Abstract

**Background:**

Toll-like receptor 4 (TLR4) ligands such as lipopolysaccharide (LPS) activate immunomodulatory functions and the migration of human mesenchymal stromal cells (hMSCs). Here, we study the migration-related gene expression of LPS-stimulated hMSCs and the role and regulation of one of the upregulated genes, encoding the interferon-induced transmembrane protein 1 (IFITM1).

**Methods:**

Gene expression profiles were determined by whole-transcriptome analysis (RNA-seq) and quantitative real-time PCR (qRT-PCR). Bioinformatics approaches were used to perform network and pathway analyses. The cell migration-related genes were identified with an in vitro wound healing assay. RNA interference (RNAi) was used to suppress the *IFITM1* gene expression. The *IFITM1* gene enhancer was analyzed by chromatin immunoprecipitation (ChIP) sequencing, ChIP-to-PCR, luciferase reporter assays, and qRT-PCR for enhancer RNAs (eRNAs).

**Results:**

RNA-seq confirmed *IFITM1* as an LPS-stimulated gene, and RNAi demonstrated its importance for the LPS-stimulated migration. LPS treatment increased the eRNA expression in enhancer region R2 (2 kb upstream) of the *IFITM1* gene and enriched R2 for H3K27ac. Bioinformatics implicated the transcription factors NF-κB and IRF1, ChIP assays revealed their binding to R2, and chemical inhibition of NF-κB and RNAi directed against IRF1 prevented R2 eRNA and *IFITM1* gene expression.

**Conclusions:**

Increased expression of the *IFITM1* gene is required for LPS-stimulated hMSC migration. We described several underlying changes in the *IFITM1* gene enhancer, most notably the NF-κB-mediated activation of enhancer region R2.

## Background

Mesenchymal stromal cells (MSCs) hold great promise for the treatment of damaged and inflamed tissues, as evidenced by a large number of ongoing clinical trials [1]. They can migrate to injury sites and promote repair by releasing growth factors or modulating immune functions [2–4]. Migrated MSCs may play beneficial immunomodulatory roles in inflammation-associated conditions such as tissue injury or infection [5, 6]. The MSCs regulate these functions through Toll-like receptors (TLRs) such as TLR4. Activation of TLR4 by lipopolysaccharide (LPS) leads to the activation of many genes, notably such including immunomodulators [6], and is orchestrated by several transcription factors (TFs), prominently NF-κB and IRF1 [7, 8].

We previously reported that TLR4 stimulation of hMSCs induced inflammatory cytokine and chemokine secretion and promoted cell migration through increased gene expression [9]. Among the cell migration-related genes, the interferon (IFN)-induced transmembrane protein 1 (IFITM1) gene was induced. IFITM1 is important for immunity, anti-viral activity, and cellular functions such as adhesion and proliferation [10]. The expression of IFITM1 is strongly induced by IFN-α and γ and mediates IFN-induced antiproliferative effects [11]. IFITM1 forms a complex with the B cell receptor [12] and is involved in inflammatory diseases such as inflammatory bowel disease [13]. IFITM1 promotes homotypic adhesion of lymphocytes and inhibits proliferation of the B cell lineage [14, 15]. In addition, IFITM1 has been implicated in the migration, invasion, and proliferation of cancer cells [16, 17] but can also inhibit tumor cell growth [18]. The present study aimed to clarify the significance of IFITM1 gene expression for LPS-stimulated hMSC migration and to characterize IFITM1 gene regulation in this context. We first characterize the transcriptomic context of the IFITM1 gene activity, which corroborates and extends our previous study [9]. We then demonstrate a requirement of IFITM1 for the LPS-stimulated hMSC migration. Further, we show the importance of both IRF1 and NF-κB for IFITM1 gene expression. Finally, we identify and characterize the responsible IFITM1 gene enhancer by profiling the IFITM gene locus for activation-related histone modifications [19–23] and enhancer RNAs (eRNAs) [24–27].

## Methods

### Cell culture of hMSCs

Human bone marrow-derived MSCs from a 21-year-old donor were purchased from Lonza [7F3915 (21-year-old female), 7F3674 (22-year-old female), and 127756 (43-year-old male), Walkersville, MD] and used at passage 5 or 6 as previously described [28]. These cells were cultured in low-glucose Dulbecco’s modified Eagle’s medium (DMEM) (Gibco, Waltham, MA) containing 10% fetal bovine serum (FBS) (Gibco) and penicillin (100 U/ml)/streptomycin (100 mg/ml) (Gibco) and cultured in a humidified incubator at 37 °C in a 95% room air/5% CO_2_ atmosphere. The medium was changed every 3–4 days. At 70–80% confluence, the cells (0.5~1 × 10^6^) were incubated with 1 μg/ml LPS (Sigma-Aldrich, St. Louis, MO) for 4 h and treated with indole-3-carbinol (I3C) (1 mM) to inhibit NF-κB.

### Transcriptomic analysis

Biological triplicate RNA sequencing was performed on independent RNA samples from control-incubated (4 h) hMSCs and TLR4-stimulated (4 h) hMSCs (1 μg/ml, three samples). The RNA-seq library was created as previously described [29]. Total RNA was extracted using RNAiso Plus (Takara, Shiga, Japan) and the QIAGEN RNeasy® Mini kit (QIAGEN, Hilden, Germany). To deplete ribosomal RNA (rRNA) from the total RNA, the RiboMinus Eukaryote kit (Invitrogen, Waltham, MA) was employed according to the manufacturer’s instructions. RNA libraries were created using the NEBNext® Ultra™ directional RNA library preparation kit for Illumina® (New England BioLabs, Ipswich, MA). Transcriptome sequencing was performed using the Illumina HiSeq2000.

For the study of differentially expressed genes, data in FASTQ files from RNA-seq experiments were clipped and trimmed of adapters, and the low-quality reads were removed by the Trimmomatic tool [30]. These FASTQ files were aligned to the UCSC hg19 reference genome with three mismatches using STAR (version 2.5.1) aligner software [31]. DESeq2 [32] was used to obtain differentially expressed genes for the default parameters. RNA-seq experiments were normalized and visualized using HOMER [33] after preparing custom tracks for the UCSC Genome Browser (http://genome.ucsc.edu/). The acquired data were deposited in the Gene Expression Omnibus database under dataset accession no. GSE81478, GSE97723, and GSE97724.

### Graphical representation of networks and pathways

To analyze the genetic networks and pathways, the RNA-seq dataset was used at a cutoff of fold change (≥ 0.8 log_2_ fold change, *P* value ≤ 0.05) in TLR4-stimulated hMSCs. These data were each mapped to objects in the Ingenuity Knowledge Base Ingenuity Pathway Analysis (IPA, Ingenuity W Systems, Mountain View, CA). The IPA software represented functional analysis that showed genes involved in biological functions and disease.

### Functional annotation

Database for Annotation, Visualization, and Integrated Discovery (DAVID), version 6.8, was used for analyzing the functional annotation in biological processes [34]. These data were used in a modified Fisher’s exact *P* value in the DAVID program, and *P* values less than 0.001 were considered significant.

### Quantitative reverse transcription polymerase chain reaction

Total RNA extraction was performed using RNAiso Plus (Takara) according to the manufacturer’s instructions. RNA samples were reverse-transcribed into cDNA using PrimeScript reverse transcriptase. The synthesized cDNA was amplified using SYBR Premix. Quantitative PCR was performed using an ABI 7500 real-time PCR system (Applied Biosystems Inc., Waltham, MD). The Ct value was normalized to glyceraldehyde-3-phosphate dehydrogenase (GAPDH) levels as an internal control. The specific primers were designed using Primer Bank (http://pga.mgh.harvard.edu/primerbank/index.html). The primers for qRT-PCR and eRNA expression are listed in Table 1 and Table 2, respectively.

**Table 1:** List of primers used in qRT-PCR studies

Gene symbol	Forward sequence (5′-3′)	Reverse sequence (5′-3′)
*CCL2*	CAGCCAGATGCAATCAATGCC	TGGAATCCTGAACCCACTTCT
*CXCL10*	GGAAGGTTAATGTTCATCATCCTAAGC	TAGTACCCTTGGAAGATGGGAAAG
*GAPDH*	AAGGTCGGAGTCAACGGATT	CTCCTGGAAGATGGTGATGG
*IFIH1*	TCGAATGGGTATTCCACAGACG	GTGGCGACTGTCCTCTGAA
*IFIT2*	AAGCACCTCAAAGGGCAAAAC	TCGGCCCATGTGATAGTAGAC
*IFITM1*	TCAACATCCACAGCGAGACC	TGTCACAGAGCCGAATACCAG
*IFITM2*	CATCCCGGTAACCCGATCAC	CACGGAGTACGCGAATGCTA
*IFITM3*	CATCCCAGTAACCCGACCG	TGTTGAACAGGGACCAGACG
*IFITM5*	CACTTGATCTGGTCGGTGTTC	CAACCACCTTCTGATCTCGGG
*IRF1*	ATGCCCATCACTCGGATGC	CCCTGCTTTGTATCGGCCTG
*NFKB1*	GAAGCACGAATGACAGAGGC	GCTTGGCGGATTAGCTCTTTT
*NFKB2*	ATGGAGAGTTGCTACAACCCA	CTGTTCCACGATCACCAGGTA
*RELB*	CCATTGAGCGGAAGATTCAACT	CTGCTGGTCCCGATATGAGG

**Table 2:** List of primers used in eRNA studies

Name	Forward sequence	Reverse sequence
IFITM1_R1_S1	CCTAGAGCAGGAAATAGCGGT	TTAAATAGCGCCTGCCCCGT
IFITM1_R1_AS1	CTGGCGCTGACCGCTATTTC	AAAGACGTAGCTCCTTCTGGG
IFITM1_R1_S2	TCAGAATCTTGCATCCTCCA	CTGGCTTCTAACCTCCAAGC
IFITM1_R1_AS2	ACAAGCCCCCTTCCTACCT	CCACATGAGCAAGATTGTGG
IFITM1_R1_S3	ACTGCAGCCTGAGGAAAGAG	CAGGACAATCCCCCAAAAG
IFITM1_R1_AS3	AGTGTCTCCTGGTGCAGGTC	GCCTAGGTCATCCAGCACTC
IFITM1_R1_S4	CTGTGTTGGGGGTCTCCAC	CCTGGGCACCACAGTGAG
IFITM1_R1_AS4	CAGGACCCCACACCACTAAC	AAATCGGGATTTGGCCGGG
IFITM1_R1_S5	TTTCACTTCCAGCCGTTAAAA	GGTGACTTTGGACTCAGCAG
IFITM1_R1_AS5	CTGGAAGAGGTGGCAGTGAT	TGGGCAAACCAAGACTTTTC
IFITM1_R1_S6	GGCAAGGACGGTTGACTTCT	GAGTGTCCAGGAAGCTGCG
IFITM1_R1_AS6	GAGTGTCCAGGAAGCTGCG	AGGCAAGGACGGTTGACTTC

### Enzyme-linked immunosorbent assay

After LPS treatment, the concentrations of CCL2 and CXCL10 in the culture supernatants were determined using human CCL2 and CXCL10 ELISA kits (Komabiotech, Seoul, Korea) according to the manufacturer’s instructions.

### Western blotting

Total protein from hMSCs was extracted using RIPA buffer [50 mM Tris-Cl, pH 7.5; 150 mM sodium chloride; 0.5% sodium deoxycholate; 0.1% sodium dodecyl sulfate; 1% (v/v) Nonidet P-40] supplemented with complete EDTA-free Protease Inhibitor Cocktail (Roche Diagnostics, Basel, Switzerland). The extracted protein was separated on SDS-polyacrylamide gels and transferred to the PVDF membranes. Western blotting was performed using anti-IFITM1 (Abcam, Milton, Cambridge, UK: ab224063.) and anti-β-ACTIN (Sigma-Aldrich, St. Louis, MO: A-5316).

### Immunocytochemistry

The cells were seeded onto coverslips in 4-well plates. After 24 h, the cells were treated with either LPS, IRF1 siRNA, or IFITM1 siRNA; washed with PBS; and then fixed with 4% paraformaldehyde. Next, the cells were treated with cold methanol for 5 min and blocking solution (5% BSA in PBS) for 1 h. The cells were then incubated with the primary antibody anti-rabbit IFITM1 (1:200, Abcam, Cambridge, UK) and the secondary antibody donkey anti-rabbit IgG (Jackson Laboratory, West Grove, PA). Thereafter, the cells were washed with PBS, mounted with a 4′,6-diamidino-2-phenylindole (DAPI)-containing mounting solution (Vectashield, Vector Laboratories, Burlingame, CA), and imaged by microscopy (Nikon Eclipse 80i Tokyo, Japan).

### Chromatin immunoprecipitation PCR and ChIP-seq

ChIP experiments were performed as previously described [9]. The ChIP-PCR assay was performed using antibodies against CTCF (Merck Millipore, Billerica, MA, 07-729), H3K27ac (Abcam, Cambridge, UK, ab4729), IRF1 (Cell Signaling, Danvers, MA; #8478), and NF-κB p65 (Abcam, ab7970). The relative enrichment levels were normalized to the input DNA for ChIP-PCR. The primers used for ChIP-PCR are listed in Table 3.

**Table 3:** List of primers used in ChIP-PCR studies

Name	Forward sequence (5′-3′)	Reverse Sequence (5′-3′)
IFITM1_R1	CGTGTCCTTCTGTGTCAGGTT	AAAAGACGCGGAAGAATTGA
IFITM1_R2	TGCCAGCTGATCCATTGAGT	TTTATAGATCAAGGCTGAACAAGC
IFITM1_R3	ACTGGGAGGTTTAGTCCCCA	AATCCCCCAAAAGCCCTCAC
IFITM1_R5	TTTCACTTCCAGCCGTTAAAA	GGTGACTTTGGACTCAGCAG
IFITM1_R6	CCCCGTCCCGAACACAAAG	TCTCCGTTTCCCCGAAGTG

The library for ChIP sequencing (ChIP-seq) was constructed using the NEBNext® Ultra™ DNA library preparation kit for Illumina® (New England BioLabs, Ipswich, MA). ChIP-seq was performed using the Illumina HiSeq2000. ChIP-seq experiments were normalized and visualized using HOMER [33]. The sequenced reads were aligned to hg19, and enriched peaks were displayed in the USCS Genome Browser.

### Wound healing migration assay

The cells were seeded into wells of a wound healing assay kit (Ibidi, Martinsried, Germany) as previously described [28]. After seeding, hMSCs were incubated for 1 day. Then, the insert was removed and hMSCs were incubated for 24 h. The cells were then visualized using a microscope (Leica, Wetzlar Germany).

### Knockdown of gene expression by siRNA treatment

Small interfering RNA (siRNA) was purchased from Ambion Applied Biosystems (Waltham, MA) according to the manufacturer’s instructions. Knockdown of IRF1 was performed using siRNA sense strand 5′-GCAGAUUAAUUCCAACCAAtt-3′ and antisense strand 5′-UUGGUUGGAAUUAAUCUGCat-3′ (ID # s7502). Knockdown of *IFITM1* was performed using siRNA sense strand 5′-CUGUGACAGUCUACCAUAUtt-3′ and antisense strand 5′-AUAUGGUAGACUGUCACAGag-3′ (ID # s16193). After seeding of hMSCs, transfection was performed using siPORT™ NeoFX™ transfection agent (Ambion Applied Biosystems; L/N: 1203023) with siRNA constructs and scrambled siRNAs (Ambion Applied Biosystems). IFITM1 and IRF1 siRNA were incubated at a concentration of 100 nM for 48 h.

### Luciferase reporter assay

Enhancer regions (R2 and R5) and promoter regions (R3) were amplified using LongAmp® *Taq* 2X Master Mix (New England BioLabs). Promoter regions were amplified using forward and reverse primers to generate *Bgl*II and *Hin*dIII sites, respectively. These constructs were cloned into pGL4.17 (Promega, Fitchburg, WI). The construct of promoter+pGL4.17 was used as a control. Enhancer regions were digested with *Kpn*I and *Xho*I. These constructs were cloned into the promoter+pGL4.17 construct. The primers used for cloning are listed in Table 4.

**Table 4:** List of primers used in the luciferase reporter assay

Name	Forward sequence (5′-3′)	Reverse sequence (5′-3′)	Location	Size (bp)
R1 (*Kpn*1-*Xho*1)	AAAggtaccTCTATCCGTCAGACAGAAACTCC	ATActcgagGGACACTATCAAGGGGCTGA	chr11:269,146–269,939	794
R2 (*Kpn*1-*Xho*1)	AAAggtaccGCCAGCTGATCCATTGAGTA	ATActcgagTTTATAGATCAAGGCTGAACAAGC	chr11:311,846–312,080	235
R3 (promoter, *Bgl* ll-*Hin*d III)	ATAagatctAACACACTACCCTCTGGGGA	ATAaagcttGTTTTCTGCGTGGAGCGAAG	chr11:313,486–314,122	637
R6 (*Kpn*1-*Xho*1)	AAAggtaccAGGGAACAGATGGCTCAGAA	ATActcgagTCTCCGTTTCCCCGAAGTGA	chr11:355,400–356,431	1032
R6 (*Xho*1-*Kpn*1)	ATActcgagAGGGAACAGATGGCTCAGAA	AAAggtaccTCTCCGTTTCCCCGAAGTGA	chr11:355,400–356,431	1032

The cells were seeded into 24-well plates and transfected with Lipofectamine 3000 (Thermo Fisher Scientific, Waltham, MA). Luciferase activity was measured using the Dual-Glo® Luciferase Assay kit (Promega, Fitchburg, WI) according to the manufacturer’s instructions. phRL (Renilla luciferase expression construct; Promega) was used as an internal control. Luciferase activity was normalized to Renilla luciferase and the control construct (promoter+pGL4.17).

### Statistical analysis

Results are given as the means ± standard deviation of the mean (SD). Data were analyzed with SPSS 17.0 (SPSS Inc., Chicago, IL) using one-way ANOVA, followed by Tukey’s honestly significant difference (HSD) post hoc test. *P* values < 0.05 were considered significant.

## Results

### Differentially expressed genes of TLR4-stimulated hMSCs

**Fig. 1:**
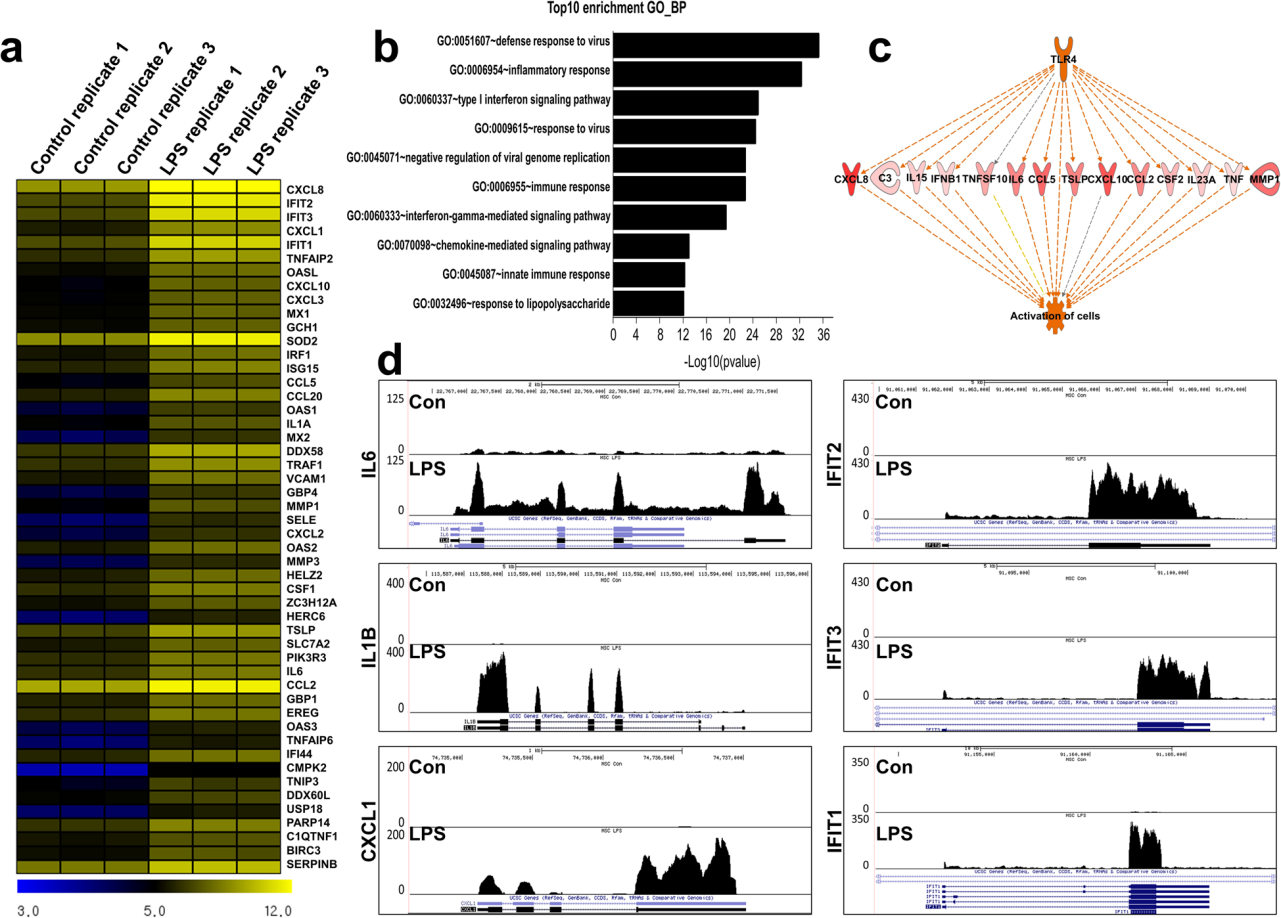
Differentially expressed genes in LPS-treated hMSCs. **a** Heat map of the top 50 upregulated genes after treatment with 1 μg/ml LPS. Each row reveals the relative expression levels of a single gene, and each column reveals the expression levels of a single sample. Each condition was performed in biological triplicate (*n* = 3). **b** Gene Ontology analysis using genes upregulated after treatment with LPS. **c** IPA regulator effect analysis identified effects on cell activation through TLR4 in LPS-treated hMSCs. **d** UCSC Genome Browser images showing normalized RNA-seq read densities in control and LPS-treated hMSCs

We started by corroborating and extending our previous transcriptome analysis of LPS-stimulated hMSCs (10 ng/ml) [9], now also including samples treated with 1 μg/ml. No morphological changes were noticed during the 4-h treatment (Additional file 1). Three hundred ninety-three upregulated and 36 downregulated differentially expressed genes (DEGs) were identified, slightly more than in our previous report (224/9), probably due to the higher LPS concentration used here. Of the top 50 upregulated genes (Fig. 1a), 39 (including the top 31) were among the top 50 of the previous study [9]. The top 50 upregulated DEGs encode chemokines (CXCL1, CXCL2, CXCL3, CXCL8, and CXCL10), cytokines (CCL2, CCL5, and CCL20), interferon-stimulated factors (GBP4, IFIT1, IFIT2, IFIT3, MX2, OAS1, and OAS2), and interleukins (IL6 and IL1A). Gene Ontology (GO) analysis using DAVID revealed that the upregulated genes were involved in biological processes (BP) such as negative regulation of viral genome replication and type I interferon signaling (Fig. 1b). IPA identified 149 potential regulators including the TLR4 receptor and linked TLR4 with inflammation-related gene products such as CXCL8, C3, IL15, IFNB1, TNFSF10, IL6, CCL5, TSLP, CXCL10, CCL2, CSF2, IL23A, TNF, and MMP1 (Fig. 1c). Furthermore, normalized RNA-seq read densities of inflammation-related (IL6, IL1B, and CXCL1) and interferon-related genes (IFIT1, IFIT2, and IFIT3) were increased in LPS-treated hMSCs (Fig. 1d); IFIT1, IFIT2, and CXCL1 had previously been studied at the lower (10 ng/ml) LPS concentration and now (1 μg/ml) yielded essentially identical density patterns.

### IFITM1-mediated migration responses in TLR4-stimulated hMSCs

**Fig. 2:**
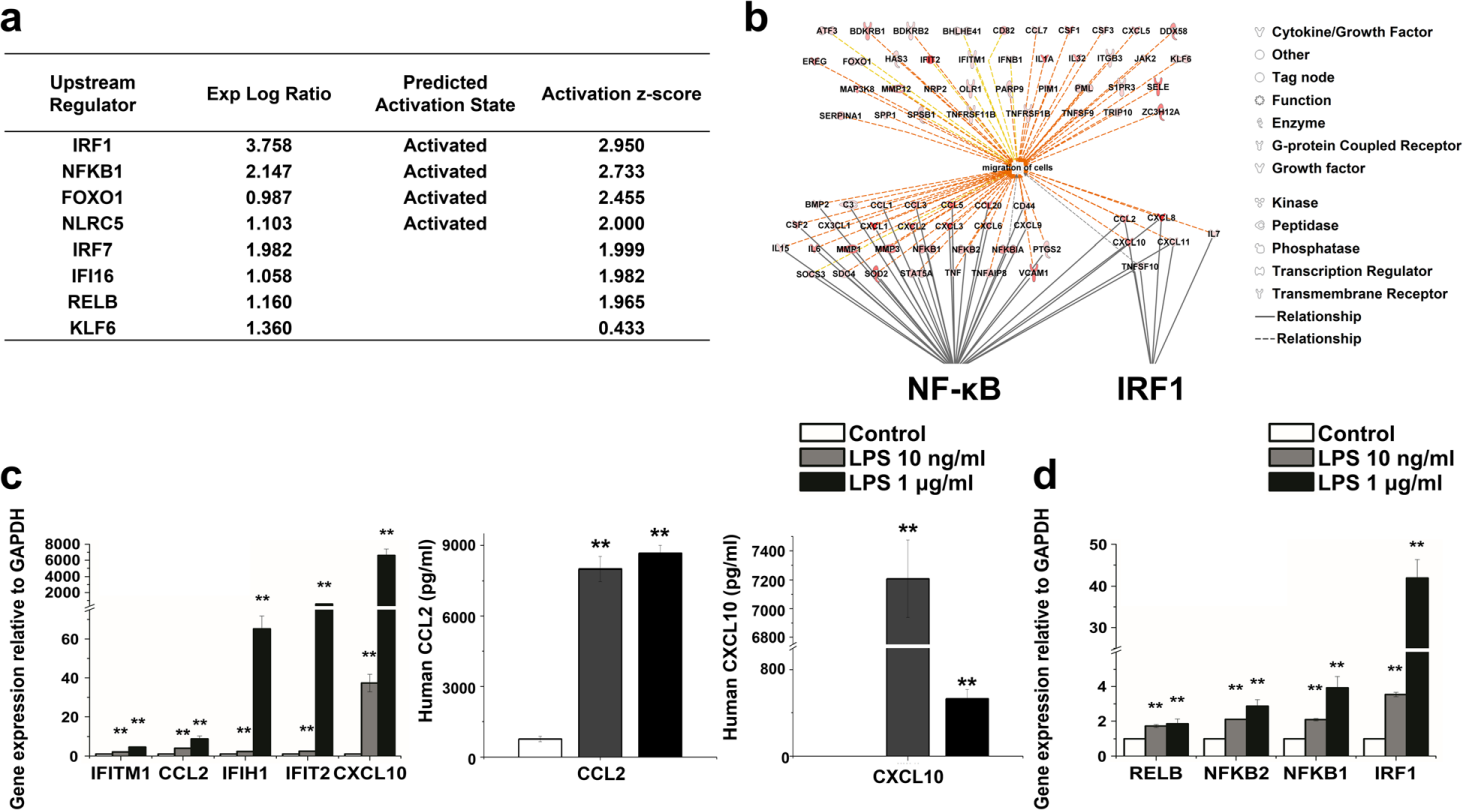
Transcriptomic analysis of TFs in LPS-treated hMSCs. **a** Upstream regulator analysis predicted the activation state of TFs including IRF1, NF-κB, FOXO1, and NLRC5 in LPS-treated hMSCs. **b** The migration-related molecules were highly correlated with NF-κB and IRF1. These molecules are presented using the IPA molecule activity predictor. **c** Confirmation of the expression levels of the cell migration-related genes using quantitative real-time PCR (left). Gene expression was normalized to GAPDH transcript levels. ELISA results showing the release of CCL2 and CXCL10 upon TLR4 stimulation of hMSCs (right). The data represent three independent experiments. **P < 0.005. **d** Confirmation of the expression levels of TFs NF-κB complex and IRF1 using quantitative real-time PCR

To further functionally characterize the TLR4-stimulated hMSCs, we performed a network analysis using IPA software. The upstream regulator analysis identified 18 upregulated transcription regulators, of which the top ones were the TFs IRF1, NFKB1, FOXO1, and NLRC5 (Fig. 2a). IPA analysis then indicated that NF-κB and IRF1 formed a direct or indirect network with several upregulated genes that were mainly involved in cell migration (Fig. 2b). The upregulation of cell migration-related genes (CCL2, CXCL10, IFIH1, IFIT2, IFITM1) was confirmed using qRT-PCR (Fig. 2c left) and, for CCL2 and CXCL10, by ELISA (Fig. 2c, right). By qRT-PCR, we demonstrated the increased expression of the TFs IRF1, NF-κB1, NF-κB2, and RELB (Fig. 2d).

**Fig. 3:**
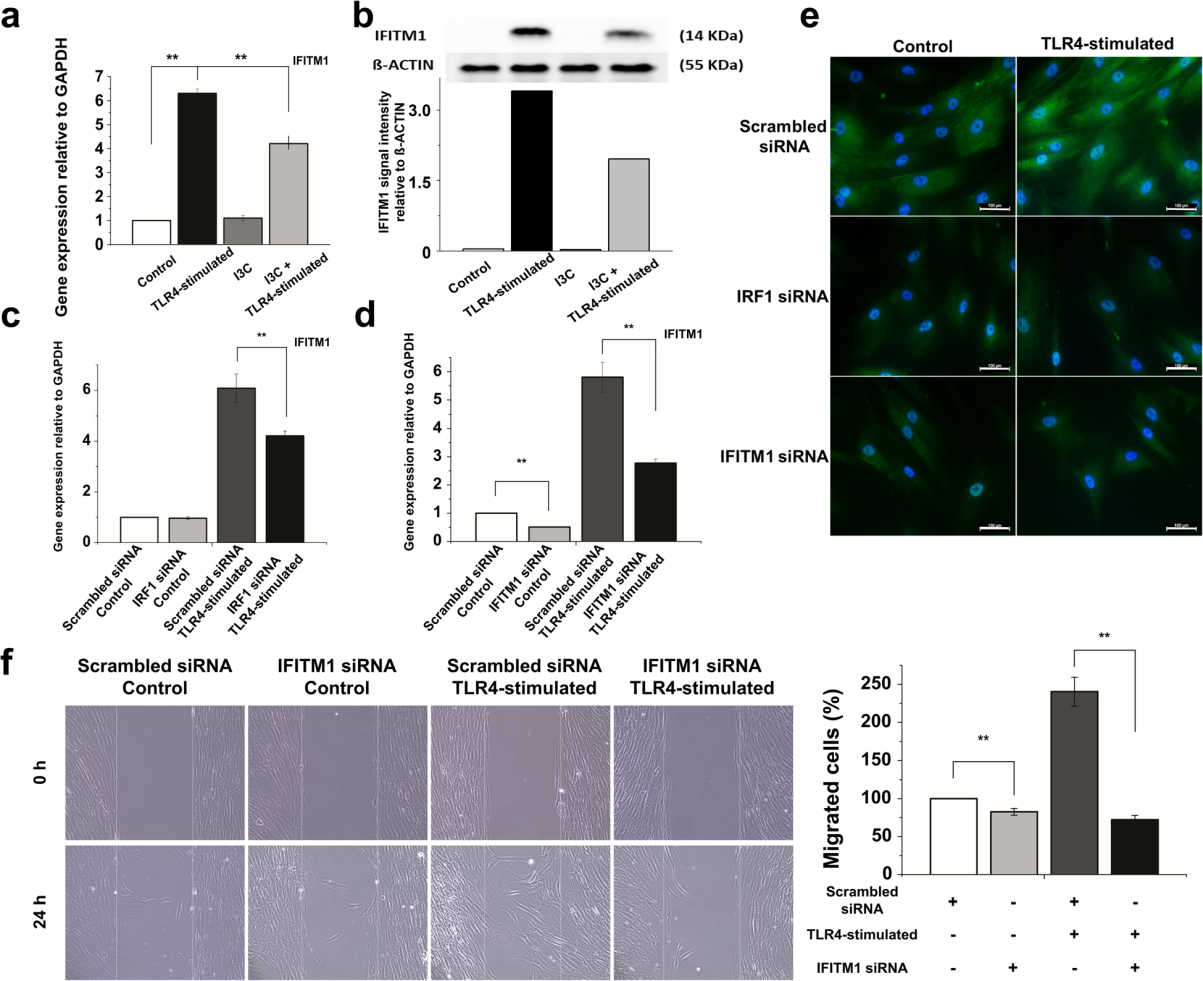
IFITM1 regulation in cell migration responses. **a** Effect of I3C on IFITM1 mRNA expression in TLR4-stimulated hMSCs measured by quantitative real-time PCR. Gene expression was normalized to GAPDH transcript levels. The data represent three independent experiments. **P < 0.005. **b** Effect of I3C on IFITM1 protein expression in TLR4-stimulated hMSCs measured by western blotting. Protein expression was normalized to β-ACTIN expression level. **c**, **d** Quantitative real-time PCR analysis of IFITM1 mRNA in IRF1 siRNA-treated hMSCs and IFITM1 siRNA-treated cells. Gene expression was normalized to GAPDH transcript levels. **P < 0.005. **e** Effect of knockdown of IRF1 and IFITM1 on the IFITM1 expression in TLR4-stimulated hMSCs assessed by immunofluorescence microscopy. Original magnification, × 400. **f** The migration of hMSCs was determined using a wound healing migration assay. Representative images of migrating hMSCs after 24 h in TLR4-stimulated or IFITM1 siRNA-treated hMSCs are shown on the left. On the right, the numbers of cells (%) migrated into the middle blank fields after 24 h of incubation are shown. There was a significant difference between the TLR4-stimulated and IFITM1 siRNA-treated hMSCs in the wound healing migration assay when compared with the TLR4-stimulated hMSCs (**P < 0.005)

Of note, the above list of migration-related genes (Fig. 2b, c) confirmed the previously published [9] induction of IFITM1, which we selected as the focus for the remainder of this study (see Background). We first tested whether NF-κB and IRF1 play a role in LPS-induced IFITM1 expression. Indeed, both inhibition of NF-κB with I3C and knockdown of IRF1 using siRNA decreased the expression of IFITM1 mRNA and protein in hMSCs stimulated with a high dose of LPS (Fig. 3a–c, e; Additional file 1: Figure S2a). We did not obtain significantly different results with the MSC samples that we used for this study and that spanned a donor age range from 21 to 43 years (data not shown). We then asked whether IFITM1 plays a role in TLR4-stimulated hMSC migration. To this end, we applied RNA interference to an in vitro wound healing assay. LPS treatment increased the number of hMSCs that migrated into the wound field within 24 h as expected, but an IFITM1 siRNA (unlike a scrambled control) suppressed IFITM1 mRNA and protein expression (Fig. 3d, e) and prevented migration (Fig. 3f). Together, these data strongly suggest that IFITM1 is regulated by NF-κB and IRF1 and plays a role in the TLR4-stimulated cell migration response of hMSCs.

### Defining the IFITM1 enhancer sites

**Fig. 4:**
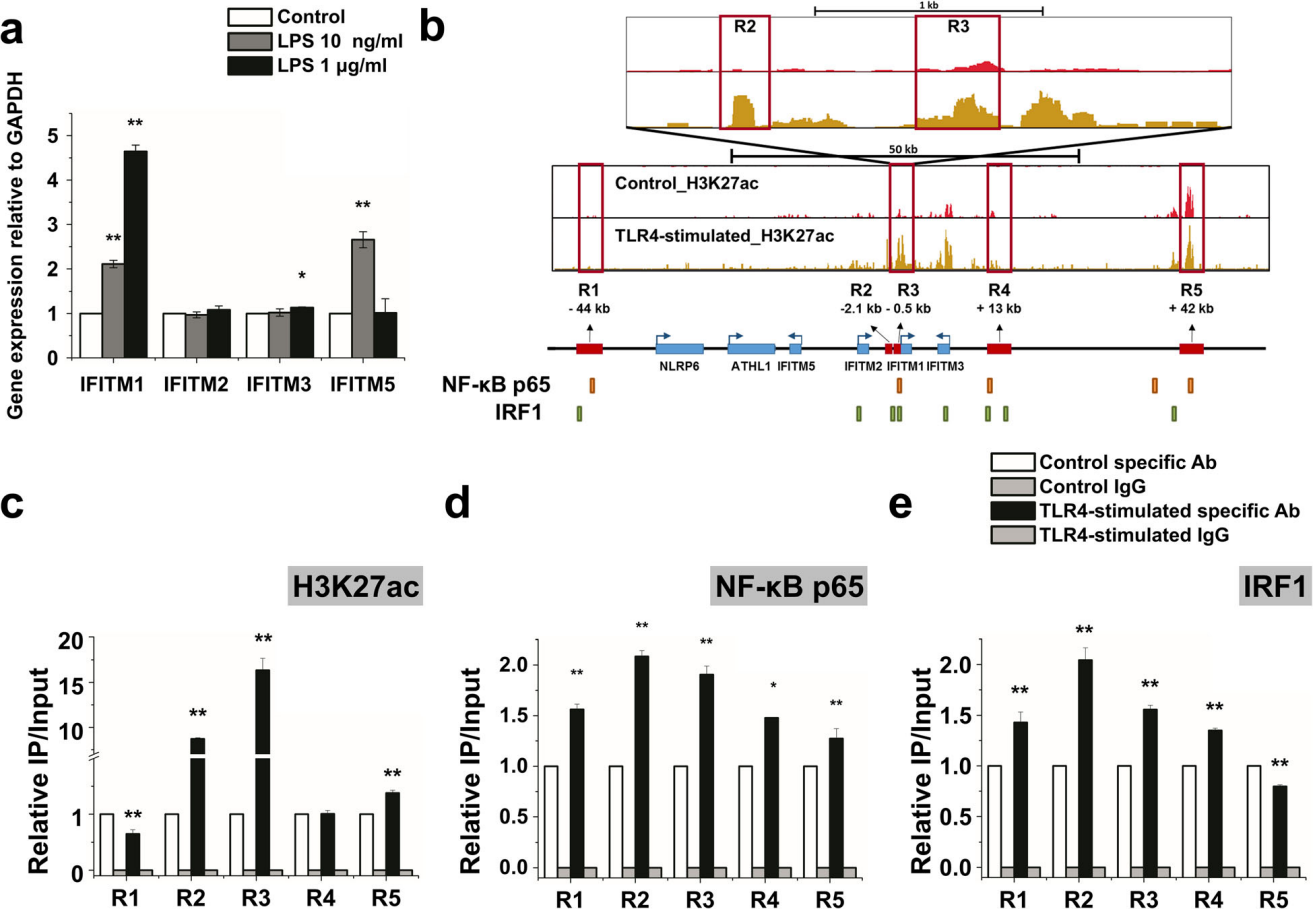
Enrichment of H3K27ac and binding of TFs in the enhancer sites. **a** Quantitative real-time PCR analysis of mRNA levels of the IFITM family after treatment with a low or a high LPS concentration. Gene expression was normalized to GAPDH transcript levels. The data represent three independent experiments. **P < 0.005. **b** Profile of H3K27 enrichment along the IFITM locus. Blue boxes indicate each gene; red boxes indicate enhancer regions. Upstream (R1, R2, R3) and downstream (R4, R5) enhancer regions of the IFITM1 gene are numbered. Orange boxes show the NF-κB binding sites; green boxes show the IRF1 binding sites (modified data from the TF search analysis of the UCSC Genome Browser using published ChIP-seq data from ENCODE). **c** Enrichment of H3K27ac on IFITM1 enhancer regions using ChIP-PCR. Enrichment was calculated relative to control input DNA from three independent experiments. *P < 0.05, **P < 0.005. **d** Enrichment of NF-κB on IFITM1 enhancer regions using ChIP-PCR. **e** Enrichment of IRF1 on IFITM1 enhancer regions using ChIP-PCR. **d**, **e** Enrichment was calculated relative to control input DNA from three independent experiments. **P < 0.005

Next, we investigated whether TLR4-stimulated IFITM1 expression is unique in the IFITM family, which contains 4 genes (IFITM1, IFITM2, IFITM3, and IFITM5). As before, the expression of the IFITM1 gene was dose-dependently increased in TLR4-stimulated hMSCs; in contrast, the expression of the IFITM2 and IFITM3 genes was not changed. The expression of the IFITM5 gene was only increased under low-dose treatment (Fig. 4a).

We further analyzed the modification state of IFITM gene-associated H3K27 by a chromatin immunoprecipitation (ChIP) assay with antibodies against acetylated H3K27. We found that the upstream regions of IFITM1 and IFITM3 were more enriched for H3K27ac in TLR4-stimulated hMSCs than in control hMSCs. Using the TF search analysis with the UCSC Genome Browser ENCODE ChIP-seq data, we evaluated the IFITM locus for potential binding sites of NF-κB and IRF1. We found five potential enhancer sites upstream (R1, R2, and R3) and downstream (R4 and R5) of the IFITM1 gene based on the enrichment of H3K27ac and binding of NF-κB and IRF1 (Fig. 4b).

We then compared the H3K27acetylation state with the actual TF binding by ChIP-PCR. This analysis showed that R2, R3, and R5, but not R1 or R4, were significantly enriched with H3K27ac in TLR4-stimulated hMSCs (Fig. 4c). Moreover, R1, R2, and R3 were significantly increased for NF-κB (Fig. 4d), while R2 and R3 were enriched for IRF1 (Fig. 4e). Interestingly, R2 was more highly enriched for both NF-κB and IRF1 than the other regions. Hence, H3K27ac and the TFs NF-κB and IRF1 were jointly enriched in some IFITM1 enhancer regions of TLR4-stimulated hMSCs.

### Expression of eRNAs and enhancer activity in enhancer sites

**Fig. 5:**
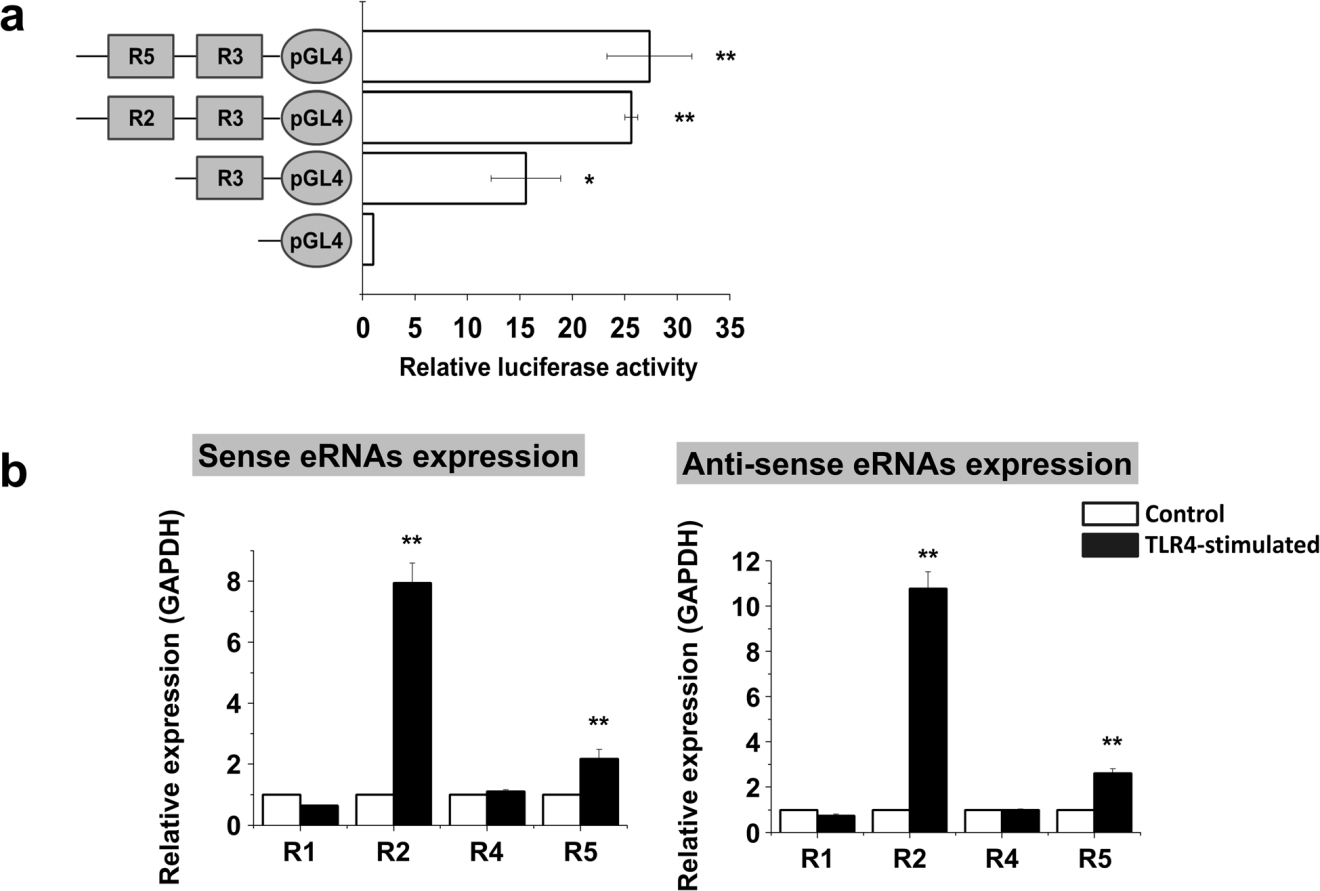
Characterization of IFITM1 enhancer regions. **a** Relative luciferase reporter activities driven by IFITM1 enhancer and promoter regions (unstimulated cells). Data are normalized to the pGL4.17 construct. The data represent three independent experiments. **P < 0.005. **b** Quantitative real-time PCR analysis of sense (**a**) and anti-sense (**b**) eRNA levels of IFITM1 enhancer regions. Expression was normalized to GAPDH transcript levels. The data represent three independent experiments. **P < 0.005

The previous results encouraged us to seek direct functional evidence for the suspected enhancer activities. To this end, we cloned the R3 promoter region (− 500 bp from the TSS of IFITM1) into the minimal promoter vector pGL4.17, immediately upstream of the luciferase gene. The R3 promoter region constructs and a no-insert control were transfected into hMSCs, and luciferase activities were measured 48 h posttransfection. There was a significant increase in the luciferase activity for R3 constructs compared with the pGL4.17 control, indicating that R3 is the IFITM1 gene promoter region (Fig. 5a). When we added either R2 or R5 to the R3-pGL4.17 construct, both caused a significant further increase in the luciferase activity.

Furthermore, we analyzed regions R1, R2, R4, and R5 for enhancer RNA (eRNA) expression. qRT-PCR detected a significant induction by LPS of both sense and anti-sense (Fig. 5b) eRNA expression in regions R2 and R5, but not R1 and R4 (since R3 showed promoter activity, we did not analyze it for eRNA expression). R2 and R5 were therefore further studied in luciferase reporter assays. Together, the eRNA and reporter gene results strongly suggest that R2 and R5 are LPS-dependent enhancers of the IFITM1 gene.

### NF-κB regulated eRNA expression in TLR4-stimulated hMSCs

**Fig. 6:**
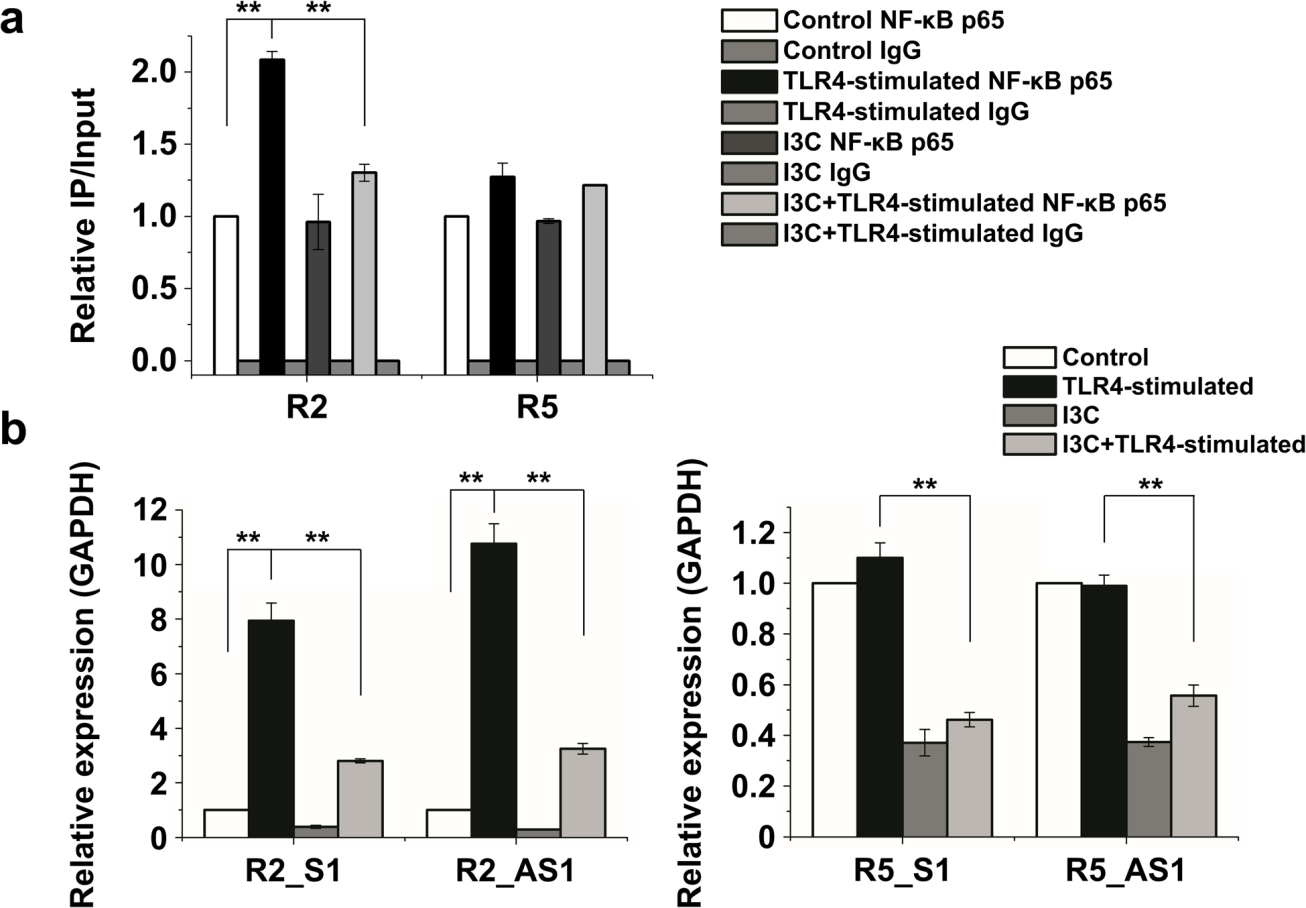
Regulation of eRNA expression by NF-κB. **a** Effects of I3C (1 mM) on the enrichment of NF-κB binding to IFITM1 enhancer regions (ChIP-PCR). Enrichment was calculated relative to control input DNA from three independent experiments. *P < 0.05, **P < 0.005. **b** Effects of I3C (1 mM) on eRNA expression of R2 and R5 in TLR4-stimulated hMSCs. The expression of eRNAs was normalized to GAPDH transcript levels. The data represent three independent experiments. **P < 0.005

Since our ChIP-PCR experiments showed NF-κB binding to R2, we tested whether R2-associated eRNA expression is regulated by NF-κB. We therefore inhibited the nuclear translocation of NF-κB with I3C (1 mM) and analyzed the resulting changes in NF-κB binding and eRNA expression by R2 and, for comparison, R5. ChIP-PCR revealed that TLR4-stimulated NF-κB binding to the R2 enhancer region was prevented by I3C (Fig. 6a). No clear effect of I3C was observed in region R5, most likely because LPS caused only a very modest increase in NF-κB binding (Fig. 6a; compare also Fig. 4d).

In contrast, regarding NF-κB binding, LPS significantly increased the levels of sense and anti-sense eRNAs corresponding to not only R2 but also R5; this was prevented by I3C (Fig. 6b). No such effect was observed for regions R1 and R4 (Additional file 1: Figure S3). These results indicate that NF-κB translocation induces eRNA expression in the enhancer and promoter regions of the IFITM1 gene.

## Discussion

The immunomodulatory and migratory properties of MSCs hold promise for therapeutic applications in tissue injury, immune-related disorders, and inflammatory conditions [35–40]. However, MSCs may also exert pro-inflammatory effects that counteract the therapeutic benefit [41]. Both the desired and undesired roles may involve stimulation of TLR4, a receptor that can be activated by LPS. Using RNA-seq, we previously reported that a low dose of LPS (10 ng/ml) upregulated inflammation-, chemotaxis-, and migration-related genes and stimulated the migration of hMSCs [9]. As the LPS concentration may be important for the net effect of TLR4 activation [42, 43], we now also tested a higher dose (1 μg/ml). Both doses upregulated genes that are associated with inflammation, chemotaxis, and cell migration, and the top 31 DEGs at 1 μg/ml were in the top 50 DEGs at 10 ng/ml. However, while a number of genes showed the highest expression at the lower LPS concentration, we observed a dose-dependent increase in IFITM1, the main focus of the present study. Therefore, we performed most experiments at 1 μg/ml LPS. Of note, only the IFITM1 gene was consistently activated by LPS in our study, contrasting, for example, with lung epithelial cells, which activate several IFITM genes (IFITM1, IFITM2, and IFITM3) through IFN-responsive enhancers [44].

Importantly, we found that RNA interference directed against IFITM1 significantly downregulated TLR4-stimulated cell migration. Thus, IFITM1 gene expression plays an important role in TLR4-stimulated hMSC migration, in line with the known pro-migratory role of IFITM1 in other cell types [15, 45]. Furthermore, the present study elucidated some of the molecular mechanisms that underlie the LPS-stimulated transcriptional activation of the IFITM1 gene.

Our ChIP-seq analysis revealed increased levels of H3K27ac upstream of the IFITM1 transcription start site (TSS). The H3K27ac-enriched regions exhibited increased binding of NF-κB (R2 enhancer and R3 promoter regions) and IRF1 (directly upstream of the TSS), in line with a previous study reporting enrichment of these TFs in LPS-induced enhancers in macrophages [7]. NF-κB and IRF1 are well-established master regulators of inflammation [46–48]. Furthermore, our results indicate that NF-κB directly bound to the IFITM1 R2 enhancer and R3 promoter regions and that this binding regulated enhancer activities and the expression of the IFITM1 gene in TLR4-stimulated hMSCs.

Most studies of the regulation of the IFITM cluster are focused on the promoter sites [49, 50], but Li et al. reported that enhancers 35 kb upstream of the IFITM3 promoter regulate the expression of IFITM1, IFITM2, and IFITM3 in interferon β-1a-treated lung epithelial cells [44]. Among the H3K27ac-enriched sites and TF (NF-κB, IRF1) binding sites flanking the IFITM cluster loci, we investigated LPS-stimulated enhancers located 2.1 kb upstream (R2 enhancer) and 42 kb downstream (R5 enhancer) of the IFITM1 gene TSS. Of these, R5 is similar to the previously described IFN-responsive enhancer located 35 kb upstream of the IFITM3 gene [44]. By contrast, the R2 region 2 kb upstream of the IFITM1 gene appears to play a role as an LPS-inducible enhancer for the IFITM1 gene (but not the other IFITM1 genes) due to the increased binding of NF-κB and enrichment of H3K27ac.

Recently, many studies reported that eRNAs are produced by RNA polymerase II [7, 24, 51]. Most of the eRNA-coding DNA sequences are transcribed bidirectionally, and the resulting eRNAs function as activators of genome-wide epigenetic mechanisms [24]. In TLR4-stimulated hMSCs, eRNA expression was significantly increased in regions R2 and R5. In addition, the activity of R3 (the promoter region) was inhibited when blocking TLR4-stimulated NF-κB nuclear translocation. Previously, NF-κB was found to bind enhancer regions and to play a role in the regulation of target gene expression [52–54]. In reporter assays, induction of RELB increased, and deletion of an NF-κB binding motif decreased, luciferase activity [52]. Additionally, NF-κB binds to enhancer regions and induces the activation of pro-inflammatory genes [53]. This previous work identified TNF-induced enhancers that are regulated by NF-κB and BRD4 [53]. However, we identified LPS-inducible enhancer regions (R2 and R5) of IFITM1 and demonstrated that the effect of R2 enhancer region activation was mediated by NF-κB binding.

Taken together, our results suggest that LPS-induced expression of IFITM1 is controlled by regions R3 (promoter) and R2 and R5 (enhancers) and that these elements are controlled by NF-κB, a known key regulator of inflammation responses in hMSCs. The remaining questions are whether the eRNAs described here directly regulate IFITM1 gene expression and whether the IFITM1 enhancers described here regulate IFITM1-mediated cell migration responses.

## Conclusion

We characterized in depth the gene expression profile associated with the migration of TLR4-stimulated hMSCs. We found that the increased expression of one of the migration-associated genes, *IFITM1*, plays an important role in this migration. We then elucidated the epigenetic and transcriptional mechanisms that are likely to regulate *IFITM1* gene expression in this context. Our results indicate that the LPS-responsive *IFITM1* gene enhancers are regulated by binding of the TFs, NF-κB, and IRF1 and by the NF-κB-stimulated expression of eRNAs in the upstream region of *IFTIM1*.

## Supplementary information

**Additional file 1: Figure S1.** No effect of TLR4 stimulation on morphology of hMSCs. Shown are control cells and treated cells with 10 ng/ml or 1 μg/ml of LPS for 4 h. Original magnifications: X100. **Figure S2.** Confirmation of knock-down efficiency of IRF1 and IFITM1 siRNA. Quantitative real-time PCR analysis of IRF1 mRNA levels in IRF1 siRNA-treated cells. Gene expression was normalized to GAPDH transcript levels. The data represent three independent experiments. **P < 0.005. **Figure S3.** Effect of I3C on eRNA expression by R1 and R4. Effects of I3C (1 mM) on eRNA expression by R1 and R4 in TLR4-stimulated hMSCs. eRNA expression was normalized to GAPDH transcript levels. The data represent three independent experiments. **P < 0.005.

## Data Availability

Raw read data of RNA-seq were deposited in the Gene Expression Omnibus database under dataset accession no. GSE81478, GSE97723, and GSE97724.

## Abbreviations

BP: Biological processes
ChIP: Chromatin immunoprecipitation
CTCF: CCCTC-binding factor
DEGs: Differentially expressed genes
eRNA: Enhancer RNA
GO: Gene Ontology
H3K27ac: Acetylation at Lys27 of the histone H3 subunit
H3K4me1: Mono-methylation at Lys4 of the histone H3 subunit
hMSCs: Human mesenchymal stromal cells
IDO: Indoleamine 2,3-dioxygenase
IFITM1: Interferon-induced transmembrane protein 1
LPS: Lipopolysaccharide
PGE_2_: Prostaglandin E2
qRT-PCR: Quantitative reverse transcription polymerase chain reaction
RNA-seq: RNA sequencing
TF: Transcription factors
TGF: Transforming growth factor
TLR: Toll-like receptor
TSS: Transcription start site
