# Supplementary material for: Epigenetic regulation of IFITM1 expression in lipopolysaccharide-stimulated human mesenchymal stromal cells
Source: Stem Cell Res Ther. 2020 Jan 7;11:16. doi: 10.1186/s13287-019-1531-3 (PMC6945778; doi:10.1186/s13287-019-1531-3)
Supplement: Supplementary file 1 — Additional file 1: Figure S1. No effect of TLR4 stimulation on morphology of hMSCs. Shown are control cells and treated cells with 10 ng/ml or 1 μg/ml of LPS for 4 h. Original magnifications: X100. Figure S2. Confirmation of knock-down efficiency of IRF1 and IFITM1 siRNA. Quantitative real-time PCR analysis of IRF1 mRNA levels in IRF1 siRNA-treated cells. Gene expression was normalized to GAPDH transcript levels. The data represent three independent experiments. **P < 0.005. Figure S3. Effect of I3C on eRNA expression by R1 and R4. Effects of I3C (1 mM) on eRNA expression by R1 and R4 in TLR4-stimulated hMSCs. eRNA expression was normalized to GAPDH transcript levels. The data represent three independent experiments. **P < 0.005. [file 13287_2019_1531_MOESM1_ESM.docx]

**Epigenetic regulation of IFITM1 expression in lipopolysaccharide-stimulated human mesenchymal stromal cells**

Sun Hwa Kim ^a^, Jin Choul Chai ^a^, Mi Ran Choi ^a^, Kyoung Sun Park ^b^, Young Seek Lee ^a^, Bert Binas ^a,*^, Kyoung Hwa Jung ^b,*^, Young Gyu Chai ^a,c,*^

^a^*Department of Molecular & Life Science, Hanyang University, Ansan, 15588, Republic of Korea*

^b^*Institute of Natural Science & Technology, Hanyang University, Ansan, 15588, Republic of Korea*

^c^*Department of Bionanotechnology, Hanyang University, Seoul, 04673, Republic of Korea*

**co-coressponding authors*


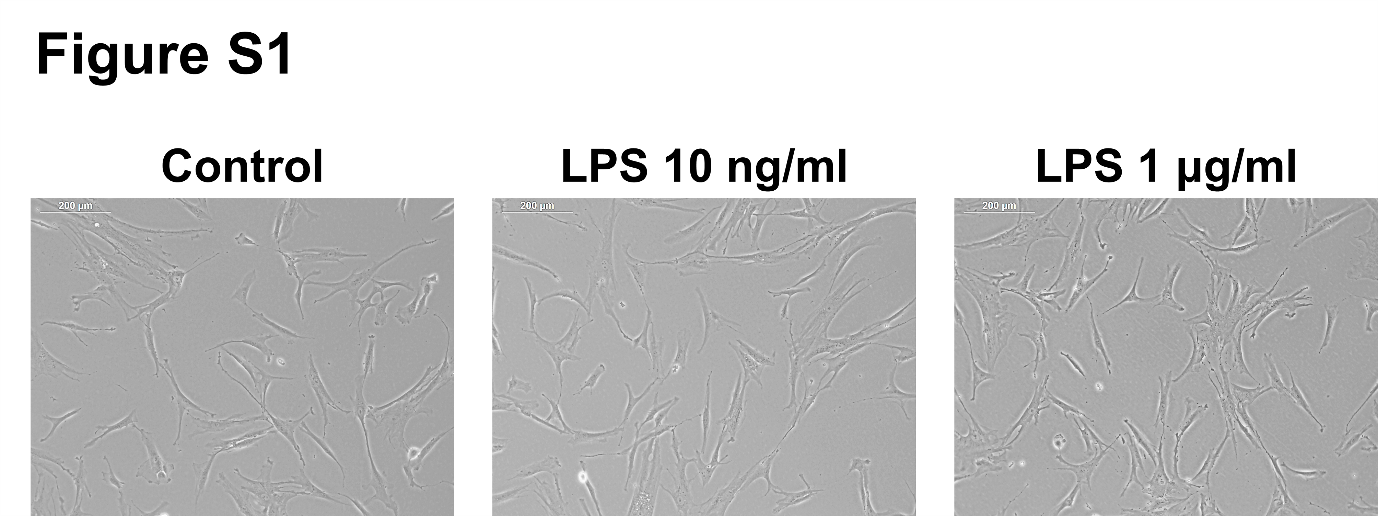


**Figure. S1.** No effect of TLR4 stimulation on morphology of hMSCs. Shown are control cells and treated cells with 10 ng/ml or 1 μg/ml of LPS for 4 h. Original magnifications: X100.


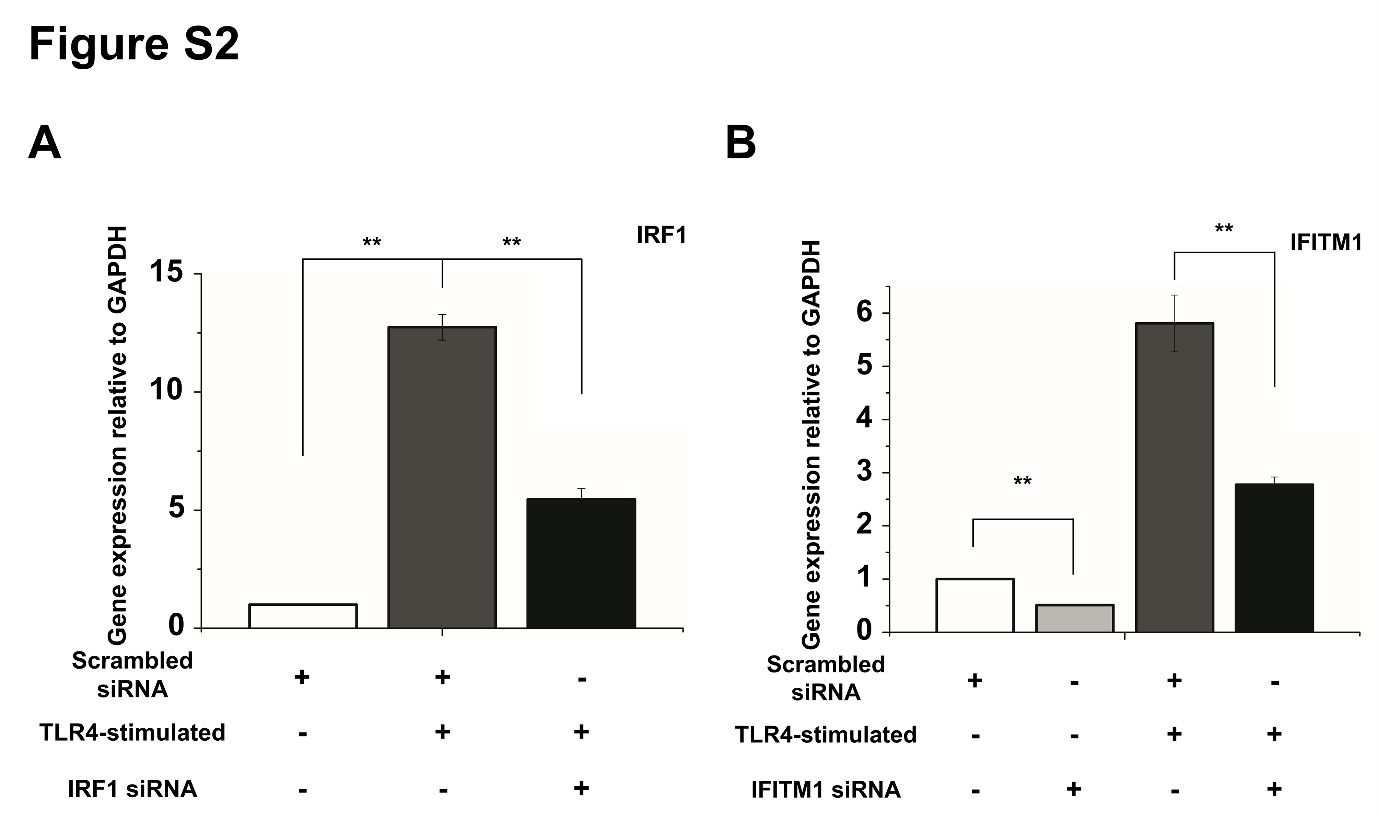


**Figure. S2.** Confirmation of knock-down efficiency of IRF1 and IFITM1 siRNA. A) Quantitative real-time PCR analysis of IRF1 mRNA levels in IRF1 siRNA-treated cells. Gene expression was normalized to GAPDH transcript levels. The data represent three independent experiments. ***P* < 0.005.


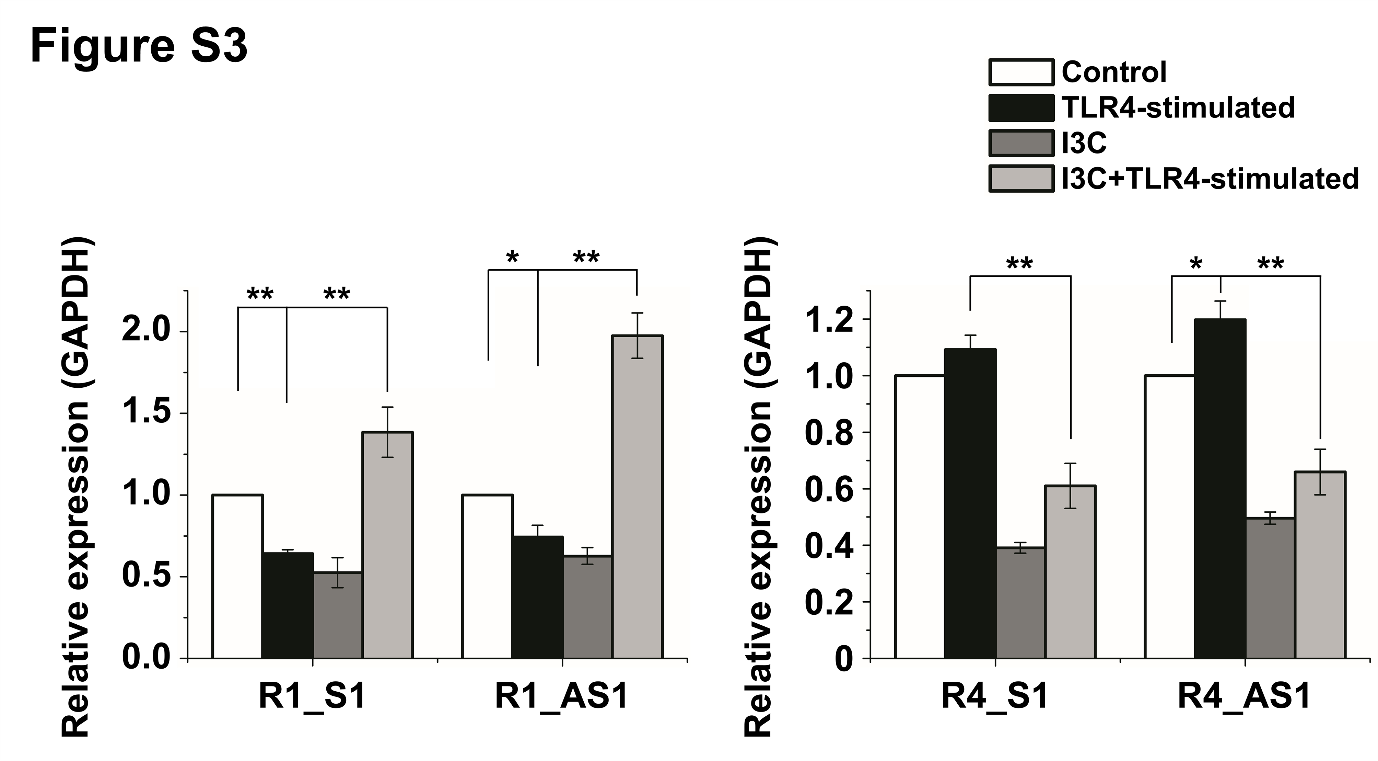


**Figure. S3.** Effect of I3C on eRNA expression by R1 and R4**.** Effects of I3C (1 mM) on eRNA expression by R1 and R4 in TLR4-stimulated hMSCs. eRNA expression was normalized to GAPDH transcript levels. The data represent three independent experiments. ***P* < 0.005.
